# Development of Organoids from Malignant Ascites for Cancer Research in Oman

**DOI:** 10.18295/squmj.6.2024.034

**Published:** 2024-08-29

**Authors:** Fazal Khan, Nausheen Yaqoob, Aida H. AlYahyaee, Shoaib A. AlZadjali, Ikram A. Burney

**Affiliations:** 1 Research Laboratories, Sultan Qaboos Comprehensive Cancer Care & Research Centre, University Medical City, Muscat, Oman.; 2 Women Health Program, Sultan Qaboos Comprehensive Cancer Care & Research Centre, University Medical City, Muscat, Oman.; 3 Gastroenterology Oncology, Sultan Qaboos Comprehensive Cancer Care & Research Centre, University Medical City, Muscat, Oman.; 4 Urogenital Cancers Program, Sultan Qaboos Comprehensive Cancer Care & Research Centre, University Medical City, Muscat, Oman.

Drug discovery and development in cancer medicine has traditionally relied on cell culture techniques (in vitro studies) and is considered a robust initial step in assessing the cytotoxic potential of novel compounds or extracts. However, limitations such as 2-dimensional cell cultures fail to replicate the tumour microenvironment accurately. The tumour microenvironment includes stromal cells, cytokine-producing cells, growth factors and metabolites, all of which influence tumour progression and growth.^1^ Therefore, the necessary next step is to assess the cytotoxic potential of selected compounds on animal models (in vivo studies). Although animal tumour microenvironments closely approximate human conditions, the procurement of laboratory animals, maintenance of animal facilities and experimentation are cumbersome, expensive and fraught with ethical issues.

More recently, organ-on-chip technology has been developed to control the cell microenvironment while maintaining tissue-specific functions.^2^ Consequently, the United States Food and Drug Administration (FDA) has removed the requirement for animal testing of new drugs, leading to a rapid expansion in the field of in vitro drug testing using organoids.^3^ Organoids are tissue-engineered 3-dimensional in vitro models that retain and recapitulate several aspects of the organ from which they are derived.^4^ Patient-derived organoids are now even employed to assess drug responses in clinical trials.^5^ The development of organoids requires specialised training and advanced modern skills. We share our experience of successful organoid development from malignant ascites of a patient with chemo-resistant high-grade epithelial ovarian cancer. To the best of the authors' knowledge, this is the first report of organoid development from Oman.

A 52-year-old female patient was diagnosed in 2023 with high-grade serous ovarian cancer, clinical stage IIIC, BRCAwt and homologous recombination proficient (HRP). She received 3 cycles of neo-adjuvant chemotherapy (paclitaxel and carboplatin) with a partial response, followed by interval debulking surgery which revealed macroscopic peritoneal metastasis (ypT3b). This was followed by another 3 cycles of chemotherapy; 3 months after completing treatment, the patient presented with abdominal distension and subacute intestinal obstruction. She was found to have a high CA 125 level, omental caking and a moderate amount of ascites. Ascitic fluid was removed and cytology confirmed disease relapse [Figure 1].

The ascitic fluid was collected for primary cell culture. The fluid was transferred into 50 mL sterile tubes and centrifuged at 1,500 × g for 15 minutes. The resulting pellet was washed with phosphate-buffered saline (PBS) and dissolved in organoid development medium (ODM), the composition of which is mentioned elsewhere.^3^ However, the medium was modified as follows: Advanced DMEM/F12 (Thermo Fisher Scientific, Waltham, Massachusetts, USA) was supplemented with GlutaMAX supplement 1X (Thermo Fisher Scientific), 10 mM Nicotinamide (Sigma Aldrich, St. Louis, Missouri, USA), 250 μg/mL Hydrocortisone (Sigma Aldrich), 10 mM HEPES (Thermo Fisher Scientific), 1.25 mM Acetyl Cystein (Sigma Aldrich), 1X B-27™ minus vitamin A Supplement (Thermo Fisher Scientific), 1X N-2 Supplement (Thermo Fisher Scientific), 10 nM 17-β Estradiol (Sigma Aldrich), EGF 5 ng/mL (Thermo Fisher Scientific), 250 ng/mL R-Spondin3 (Miltenyi Biotec, Cologne, Germany), 100 ng/mL Noggin (Miltenyi Biotec), 25 ng/mL bFGF/FGF-2 (Thermo Fisher Scientific), 10 μg/mL Human hepatocyte growth factor (HGF) (Stemcell Technologies, Vancouver, Canada), 10 μM SB202190 (Stemcell Technologies), 500 nM 83-01 (Stemcell Technologies), 10ng/ml Heregulin-beta-1 (Stemcell Technologies), 100 ng/mL recombinant human IGF-1 (Thermo Fisher Scientific), 10 μM Y-27632 (Stemcell Technologies), 1X penicillin/streptomycin, 2.50 μg/mL Amphotricin B, and 0.1 mg/ml Gentamicin. Cells were counted using an automated cell counter Countess 3 (Thermo Fisher Scientific).

For organoid preparation, 24-well plates were incubated overnight in a CO_2_ incubator at 37°C. Ascitic fluid cells in ODM were mixed with Geltrex (Thermo Fisher Scientific) in a 3:4 ratio. Approximately 40,000 cells were seeded in a dome shape and incubated at 37°C for 20 minutes to facilitate the attachment of the cells and Geltrex mixture to the plastic surface. Afterwards, 1 mL of ODM was added to each well and the cultures were maintained at 37°C under 5% CO_2_ atmospheric pressure and humidity. The medium was refreshed every 2–3 days. Organoids were imaged using Olympus Inverted Microscope IX53 [Figure 2].

For organoid passaging, the spent medium was replaced with 1 mL of pre-cooled PBS and washed twice. Organoids were detached by adding 200 μL of dispase (5 U/mL) to each well. By pipetting up and down, a single-cell suspension was collected into a 15 mL sterile tube. The cells were centrifuged at 125 × g for 5 minutes and the pellet was resuspended in PBS to proceed with immunocytochemistry.

Cells from the ascitic fluid sample and those released from the organoid were fixed in 10% formalin for 3 hours at room temperature and then embedded in paraffin. Upon deparaffinisation, 4 μm sections were stained with haematoxylin and eosin (H&E) and with p16 (Clone E6h4), CA-125 (OC125; Clone M11) antibodies and Anti-Human Epithelial Antigen (Clone BerEP4) [Figure 3]. The stained slides were evaluated for morphological features and immunocytochemical profile similarity by the surgical pathologist. Both cell sets showed expression of p16 [Figures 1B and 3B] and CA-125 [Figures 1C and 3C] and BerEP4 [Figure 1D and 3D], suggesting that the cells in the organoids originated from the malignant cells in the ascitic fluid. Verbal consent was obtained from the patient for publication of this data.

We report the development of organoids from primary cell culture. To the best of the authors' knowledge, this is the first successful development of organoids in a research laboratory in Oman. Organoids can be used not only for drug discovery, drug testing and personalised medicine but also to study carcinogenesis and specific molecular alterations.

## Figures and Tables

**Figure 1:**
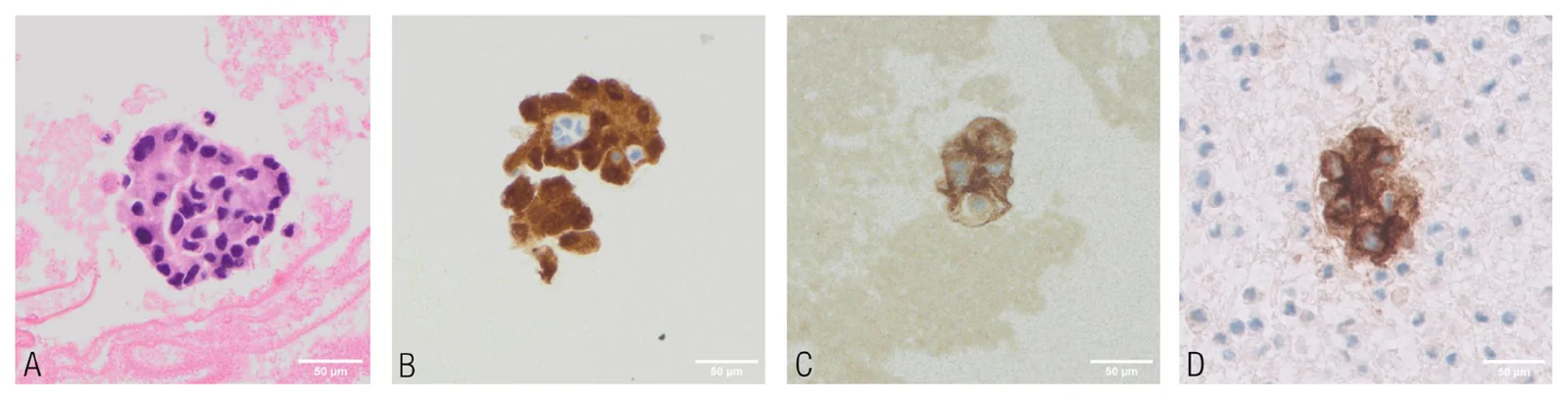
Malignant cells in ascitic fluid (at ×40 magnification) (**A**) haematoxylin and eosin stain; (**B**) p16 showing strong nuclear stain; (**C**) CA-125 exhibiting membrane staining; and (**D**) BerEp4 exhibiting membrane staining in tumour cells. *Scale bar = 50 μm*.

**Figure 2:**
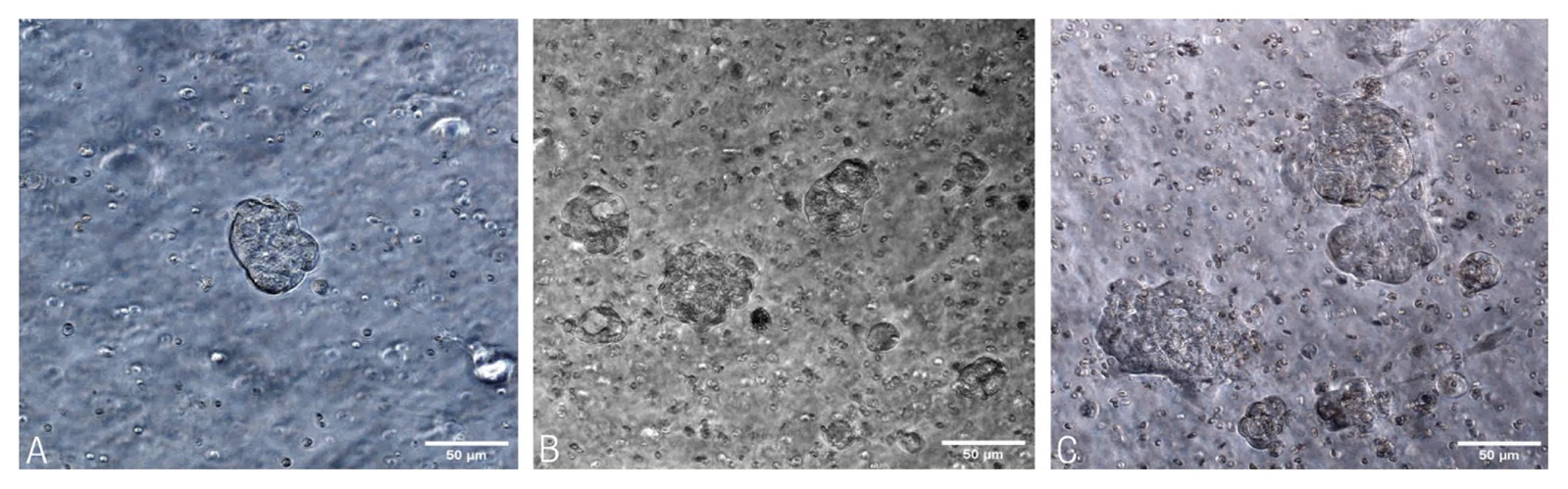
Representative phase contrast images ×20 magnification of organoids developed from the malignant ascites.

**Figure 3:**
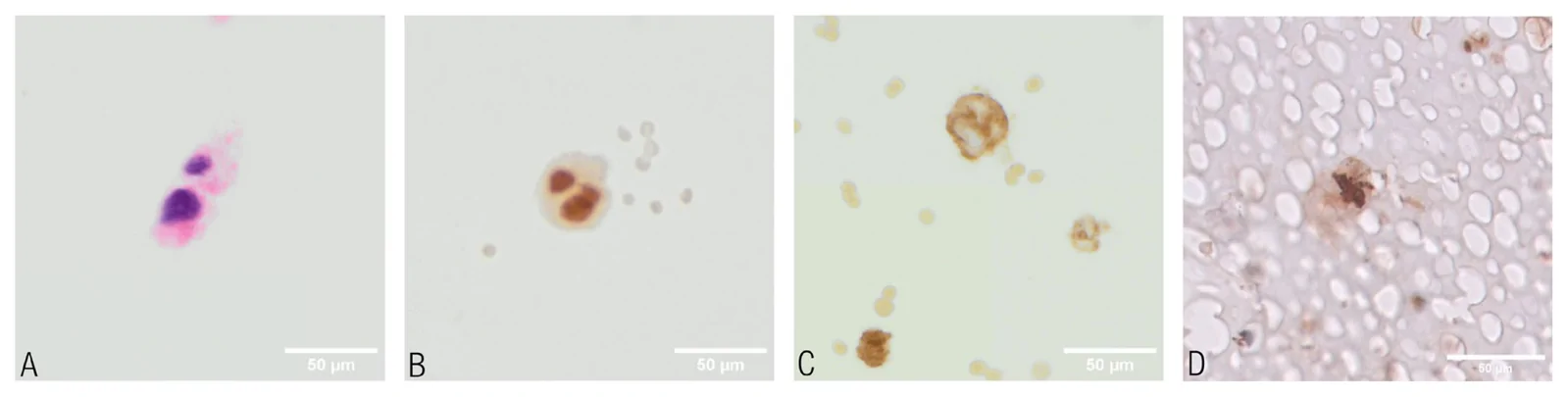
Single cell suspension of organoid (**A**) haematoxylin and eosin stain (at ×40 magnification); (**B**) p16 exhibiting strong nuclear expression; (**C**) CA-125 exhibiting membrane staining; and (**D**) BerEp4 positivity in cell suspension of organoid. *Scale bar = 50 μm*.
